# A State-of-the-Art Review for Gastric Histopathology Image Analysis Approaches and Future Development

**DOI:** 10.1155/2021/6671417

**Published:** 2021-06-26

**Authors:** Shiliang Ai, Chen Li, Xiaoyan Li, Tao Jiang, Marcin Grzegorzek, Changhao Sun, Md Mamunur Rahaman, Jinghua Zhang, Yudong Yao, Hong Li

**Affiliations:** ^1^Microscopic Image and Medical Image Analysis Group, College of Medicine and Biological Information Engineering, Northeastern University, Shenyang 110169, China; ^2^Cancer Hospital of China Medical University, Liaoning Cancer Hospital and Institute, Shenyang 110042, China; ^3^Control Engineering College, Chengdu University of Information Technology, Chengdu 610103, China; ^4^Institute of Medical Informatics, University of Luebeck, Luebeck, Germany; ^5^Shenyang Institute of Automation, Chinese Academy of Sciences, 110169, China; ^6^Department of Electrical and Computer Engineering, Stevens Institute of Technology, Hoboken, NJ 07030, USA

## Abstract

Gastric cancer is a common and deadly cancer in the world. The gold standard for the detection of gastric cancer is the histological examination by pathologists, where Gastric Histopathological Image Analysis (GHIA) contributes significant diagnostic information. The histopathological images of gastric cancer contain sufficient characterization information, which plays a crucial role in the diagnosis and treatment of gastric cancer. In order to improve the accuracy and objectivity of GHIA, Computer-Aided Diagnosis (CAD) has been widely used in histological image analysis of gastric cancer. In this review, the CAD technique on pathological images of gastric cancer is summarized. Firstly, the paper summarizes the image preprocessing methods, then introduces the methods of feature extraction, and then generalizes the existing segmentation and classification techniques. Finally, these techniques are systematically introduced and analyzed for the convenience of future researchers.

## 1. Introduction

### 1.1. Background

Cancer is a disease in which human cells grow out of control. In 2018, about 9.6 million people died of cancer [1]. Gastric cancer is a kind of cancer that is caused by an abnormal cell population that proliferates endlessly in the stomach and eventually forms tumors. According to the morphological characteristics of gastric tumors, gastric cancer can be classified into the following categories: adenocarcinoma, mucinous carcinoma, signet ring cell carcinoma, adenosquamous carcinoma, squamous cell carcinoma, and undifferentiated cell carcinoma. Adenocarcinoma accounts for more than 95% of all gastric malignancies. In general, gastric cancer refers to gastric adenocarcinoma. Adenocarcinoma includes tubular adenocarcinoma and papillary adenocarcinoma. Tubular adenocarcinoma has a well-defined glandular lumen.

Gastric cancer ranks second in morbidity and mortality among all cancers [2]. Gastric cancer kills about 800,000 people a year, according to the World Health Organization (WHO). China and Japan have the highest incidence of stomach cancer, accounting for 30 percent of all cancers, and the number of cancer cases each year is also increasing in the United States. In gender analysis, gastric cancer is the second most common cancer among men, at 26 percent, and the third most common among women, at 11 percent. It is estimated that about 30,000 new cases of gastric cancer occur each year.

The diagnosis of gastric cancer is mainly through pathological biopsy, which is stained with hematoxylin and eosin. The biopsy morphology and tissue characteristics under the microscope are observed, and the detection results are determined by a synthesis of the doctor's knowledge. But each pathology doctor's diagnosis is made according to their different experiences and states, which may result in making different judgments on gastric cancer tissue pathology images. At the same time, the pathologist has to examine a large number of histopathological images every day [3]. The diagnosis process requires long periods of concentration, and long hours of work can lead doctors to misdiagnose situations. Therefore, accurate screening and diagnosis of gastric cancer by pathologists is a major problem [4]. The number of pathologists is also very scarce. In order to alleviate the shortage of pathologists and reduce the misdiagnosis rate of histopathological examination, the CAD system is introduced into the detection of pathological images of gastric cancer [5]. With the assistance of a CAD system, the area of the malignant tumor is marked, and the accurate judgment of the computer is used as a second opinion to assist the pathologist in making a judgment [6].

The CAD system began in the 1980s. Its main purpose is to assist pathologists to make judgments by using the accuracy and efficiency of computers. The CAD system can make judgments objectively, and excellent algorithms can reduce the processing time [7]. In the past few decades, the continuous progress of machine learning algorithms has enabled the rapid development of CAD technology in gastric cancer, which can more quickly and accurately identify cancer regions [8] [9].

In the CAD system of machine learning in the field of gastric cancer, there are mainly two kinds of segmentation and classification. The main step of the classification algorithm is to preprocess the image first, in order to improve the quality of the image and make the data meet the experimental requirements. Then, feature extraction is carried out to find the features of the image that are of interest to the experiment. Finally, a suitable classifier is designed to classify the features. The segmentation algorithm consists of two steps: image preprocessing and image segmentation [10]. In recent years, a new technology has emerged: deep learning technology. A deep learning algorithm can directly act on RGB images, automatically learn the features of images through convolutional neural network, search for similar features of experimental data through a lot of training, and finally achieve segmentation and classification [11].

### 1.2. Motivation

At present, some references summarize related works of histopathological image analysis approaches, but little is done for gastric cancer. Therefore, this work focuses on the technical analysis of gastric cancer histopathological images.

In 1979, the comprehensive survey “Computer-Aided Medical Diagnosis: Literature Review” was presented [12]. This paper summarizes all the medical diagnostic techniques involved in the development, testing, and application of CAD. In this survey, there is one work related to gastric cancer.

In 2004, the paper in [13] proposed a survey paper about “Artificial Intelligence in Medicine.” In this paper, artificial intelligence is introduced that can analyze complex medical data and can diagnose, treat, and predict outcomes in the field of medicine. This paper is related to seven histopathologies, one of which is gastric histopathology.

In 2005, the paper in [14] presents a survey about “automated cancer diagnosis based on histopathological images.” In this paper, from the three aspects of image preprocessing, feature extraction, and image classification, a total of 75 papers are summarized. A total of 11 cancers are covered, but only one is related to stomach cancer.

In 2007, a survey on “Neural Networks and Other Machine Learning Methods in Cancer Research” was completed [15]. This paper summarizes some machine learning methods mainly used in the field of cancer and discusses their respective advantages and disadvantages in the field of medicine. In this work, ten types of cancer tissues are mentioned, but only one paper is about gastric cancer.

In 2018, a survey on “Deep Learning and Medical Diagnosis: A Review of Literature” was carried out. This paper provides a comprehensive analysis of deep learning techniques in histopathological images [16]. There are 46 articles on deep learning in this work, but there is only one paper that focuses on GHIA. Besides the related work mentioned above, there are some other surveys for histopathology analysis [17, 18, 19, 20], but they do not involve gastric cancer.

**Figure 1:**
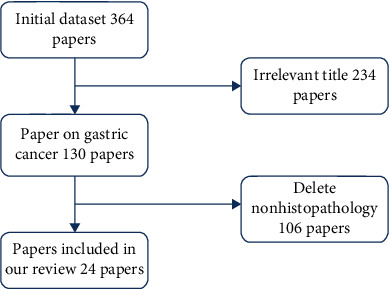
The systematic flowchart of paper selection for our work.

**Figure 2:**
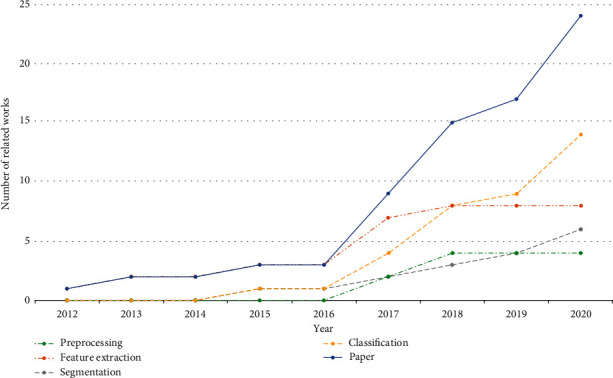
The related work presents line charts of time and quantity.

From the works mentioned above, we can find that there are many review papers on histopathological image analysis, which summarize various cancers in the medical field. However, none of them specially focus on histopathological images in the direction of gastric cancer.In this paper, we summarize a state-of-the-art review for gastric histopathology image analysis approaches.  Figure 1 shows the histopathological image literature of gastric cancer collected by Google Scholar according to the keywords of histopathological analysis of gastric cancer. A total of 364 relevant papers were downloaded, and 234 papers that were not about gastric cancer were deleted through simple reading, and then 106 papers that were not about histopathological images in gastric cancer were also deleted; finally, 24 papers were selected. As shown in Figure 2, this paper classifies and summarizes the literature according to four aspects: preprocessing, feature extraction, segmentation, and classification. As can be seen from Figure 2, papers related to GHIA were published mainly since 2012, and the number of relevant papers gradually increased after that. There are only 4 and 6 papers on image preprocessing and image segmentation, while the feature extraction method has 8 papers. There are 14 papers with the largest number of classification methods.

**Figure 3:**
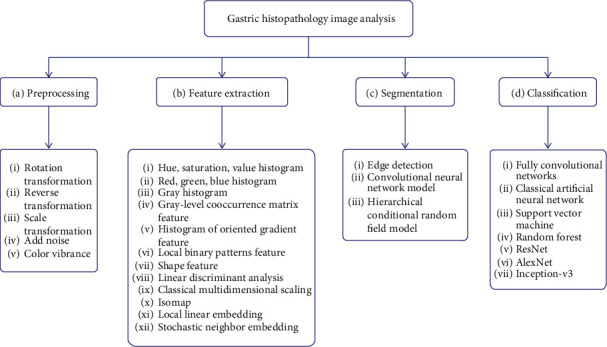
A framework for gastric histopathology image analysis.

Figure 3 is the structure diagram of this paper, summarizing the histopathology of gastric cancer from four aspects. Figure 3(a) shows the preprocessing aspect. Data enhancement is used in the preprocessing step, augmenting the dataset and preparing for the following experiments. Figure 3(b) shows the feature extraction aspect. The histograms of oriented gradient (HOG) feature and the gray-level cooccurrence matrix (GLCM) feature, along with the other features, are used for extracting the color, texture, and other characteristics of the nucleus. Linear discriminant analysis (LDA) and local linear embedding (LLE) are the processing feature vectors. Figure 3(c) shows the segmentation aspect. The minimum-model method and the deep learning network are devoted to find the nuclear region. Figure 3(d) shows the classification aspect. The artificial neural network (ANN) and the convolutional neural network (CNN) are applied to screen the cells needed for the experiment from histopathological images.

## 2. Image Preprocessing Methods

### 2.1. Related Work

Image preprocessing [21] is an important part of GHIA. In experiments, the quantity of data may be small and the image quality may be poor. Therefore, in order for experiments to be more accurately classified or segmented, a mass of datasets are needed. The experimental data need to be preprocessed so that the data can meet the experimental requirements and get better results.

In [22], a method is proposed for presenting an automatic lymphocyte detection model based on the Deep Convolutional Neural Network (DCNN) in immunohistochemical images of gastric cancer. This work extracts data from a 40-fold magnification scan of full-sized micrographs of gastric cancer tissues. The experimental data consist of 3,257 images. However, these datasets are far from enough. In the study, the data expansion methods of rotation and reflection are used to increase the number of datasets, resulting in a total of 10,868 datasets.

The work in [23] comes up with an efficient learning algorithm to replace the traditional feature extraction method. This algorithm requires a large dataset for machine learning. Some standard data augmentation methods are used to generate a large number of images, and various data enhancement methods are used to process the data in this work, including 0.3x overlap, reflection, postreflection rotation, and shearing. Through these methods, 21,000 images can be produced per pathological section, and a total of 11 sections of experimental data are generated for a total of 231,000 datasets.

The work in [24] proposes a framework for automatic recognition of gastric cancer based on deep learning. In this work, the resolution of the original gastric image dataset is 2048 × 2048, and the deep learning network cannot directly process the gastric image, so a patch of 224 × 224 is intercepted from the original gastric image. The gastric dataset contains 560 gastric cancer sections and 140 normal sections. In order to increase the dataset, the patch is rotated 90°, 180°, and 270°. After rotation, the data are cut, and the data becomes 8,992 pieces of gastric cancer data and 14,000 pieces of normal data.

In [25], an image classification model is proposed that can alleviate the bad annotation training set. By fine-tuning the neural network in two stages and introducing a new intermediate dataset, the performance of the network in image classification with a poor annotation training set is improved.

### 2.2. Summary

**Figure 4:**
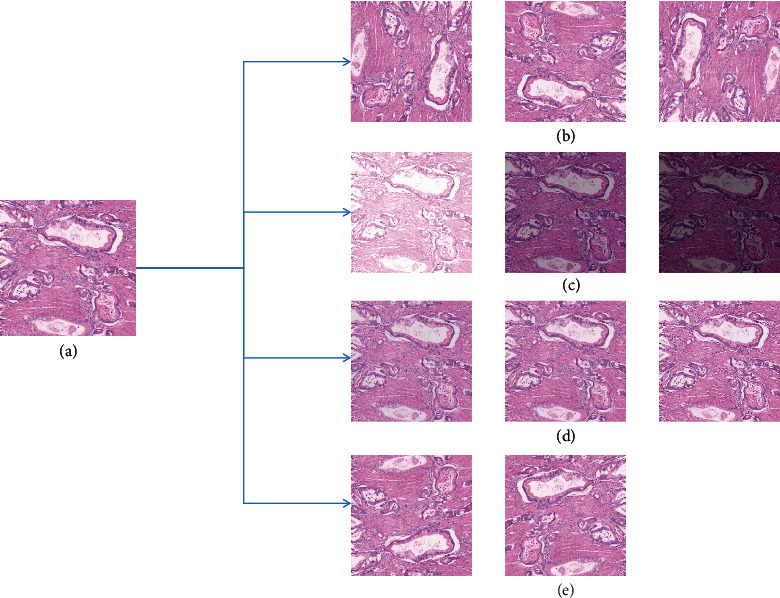
Some common augmentation approaches in the preprocessing stage.

It can be concluded from the above works that machine learning requires a huge number of datasets. Augmentation approaches are the main solution. Augmentation approaches mentioned in the above work include rotation, reverse, and scale transformation. In addition, there are some other data augmentation approaches, such as adding noise and color vibrance.  Figure 4(a) shows the original image.  Figure 4(b) shows rotation transformation, with a clockwise rotation of 90°, 180°, and 270° from the original image.  Figure 4(c) shows color vibrance, with brightness enhanced at 10% or brightness reduced to 10% and 20%. In Figure 4(d), noise is added: Gaussian noise, salt and pepper noise, and Poisson noise; Figure 4(e) shows reverse transformation: horizontal rotation and vertical rotation.

## 3. Feature Extraction Methods

### 3.1. Related Work

Feature extraction [26] refers to finding representative data in the region of interest in the image. Feature extraction mainly includes three parts: the color feature, the texture feature, and the shape feature. Some of them require postprocessing of features, such as feature dimensionality reduction.

### 3.2. Color Feature

Features based on color intensity are very important in pathology. Due to the use of special staining, the cytoplasm, nucleus, cell wall, and other stains are different in the pathological image, which can be manifested by color features. The work in [23, 27] extracts hue, saturation, and value (HSV) histograms; gray histograms; and red, green, and blue (RGB) histograms as color features to describe the difference between the colors.

### 3.3. Texture Feature

Texture feature is also a commonly used histopathological image detection method. In the histopathological images of gastric cancer, the texture features can be extracted from the nucleus and cell wall. Texture features mainly include the GLCM feature, the HOG feature, and the local binary pattern (LBP) feature.

In [23], GLCM, LBP, and Gabor filter bank features are extracted, respectively, as texture feature extraction in the comparison experiment.

In [28], the HOG feature is extracted from gastric cancer histopathological images. The HOG feature is drawn on normal, benign, and malignant gastric images to obtain the HOG feature vector. The histogram of gastric cancer is drawn by HOG feature histograms. Then, the data in the HOG histogram is directly used for classification. The accuracy rate of this work classification is 100%.

The work in [29] proposes a HOG-LDA-ANN method for gastric histopathological image classification. HOG features are compared with the GLCM and LBP features. HOG-LDA-ANN has an accuracy rate of 88.9%. There are two contrast experiments: GLCM-LPP-ANN has an accuracy rate of 85.56% and LBP-LPP-ANN has an accuracy rate of 80.12%.

The work in [30] extracts the HOG and LBP features. This work tests many feature dimension reduction methods and classifiers. Through comparison, the LBP feature is superior to the HOG feature in gastric cancer histopathological images.

### 3.4. Shape Feature

A shape feature is used to extract the topological structure information from the image as a feature. For example, the distance of some points in the image is extracted as features and then classified.

The work in [31] obtains the location information of the nucleus and then extracts features. First, the alignment and mean distance of the three adjacent cells are measured, and then the mean value and standard deviation of these two indicators are calculated as the first feature set. Another feature set is extracted from a circle of 40 microns around the central cell. The ratio of the number of nuclei in the circle to the empty circle section is calculated as the feature.

The work in [32] extracts four types of nuclear location information: epithelial cells, leukocytes, fibrocytes, and conglomerates, and obtains the cell nuclei attributed relational graph. Then, the mean, variance, skewness, and kurtosis features can be obtained from the vertices and edges of the nucleus.

### 3.5. Postprocessing Methods of Features

In [29], the HOG-LDA-ANN method for gastric cancer images is proposed. LDA is a dimension reduction technique for supervised learning, which can retain and screen effective features and eliminate inefficient features. In this work, 46,900 HOG feature vectors are obtained through feature extraction, and 90 groups of vectors are obtained through feature dimensionality reduction by the LDA algorithm.

The work in [30] compares a variety of feature reduction methods: Sammon mapping, stochastic neighbor embedding, Laplacian mapping, Isomap, classical multidimensional scaling, local linear embedding, linear discriminant analysis, and *t*-distribution random neighbor embedding. According to the final classification accuracy, two better classification methods are obtained: the LBP-MDS-ANN and LBP-LLE-ANN methods.

The work in [33] improves the PCA+LDA algorithm. Based on the traditional PCA+LDA, the LDA transformation is optimized. This method improves the generalization of traditional PCA+LDA to test samples and improves the classification accuracy. The optimized algorithm improves the classification accuracy by 3.43%.

### 3.6. Summary

This paper summarizes the feature extraction method in Table 1. In conclusion, among the three features, color features have strong limitations. It is only useful for gastric cancer histopathological images with good staining effect and high contrast. Texture features are widely used, and many articles choose the texture feature method. Shape features are used the least, and most of the extraction methods are to extract the nucleus and extract the features from the position relationship between the nuclei.

For the feature postprocessing of gastric cancer histopathology, dimensionality reduction is the main method, and features are processed by PCA, LDA, and other different algorithms. In general, LDA has a good effect on the feature dimension reduction of traditional methods. In the analysis of histopathological images of other cancers, we have also consulted relevant postprocessing methods. Although there are few relevant literatures, the main methods are PCA and LDA, such as in cervical cancer [34, 35], breast cancer [36, 37], and colorectal cancer [38].

## 4. Segmentation Methods

### 4.1. Related Work

Image segmentation [39] divides the image into several specific and unique regions. Then, the part of interest in the experiment is extracted. Image segmentation is an important step in image analysis.

In [27], the authors propose a nuclear segmentation technique, which automatically separates the nucleus by using a minimum-model method. The minimum model consists of two main steps. The first is the minimum prior information, and the second is the contour detection method independent of the image shape. This method avoids the segmentation deviation of shape features and can be accurately segmented.

**Figure 5:**
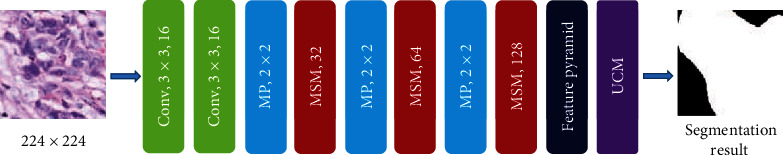
The framework of the proposed method in [40]. This figure corresponds to Figure 2 in the original paper.

In [40], the authors propose a deep learning model for the segmentation of histopathological images of gastric cancer. The workflow of this approach is shown in Figure 5. It contains two convolutional layers, three pooling layers, three multiscale module, one feature pyramid module, and one upsampling convolutional module. The convolution layer uses a3 × 3convolution kernel and the maximum pooling size is 2 × 2. In order to extract and fuse features, multiscale modules are used for the shallow layer and feature pyramids are used for the deep layer layers. The segmentation performance of the framework is 90.88%.

**Figure 6:**
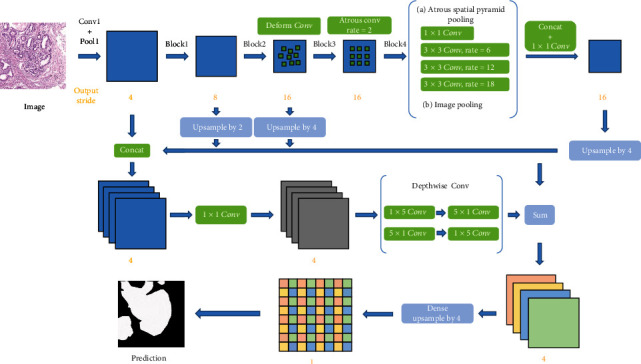
The framework of the proposed method in [41]. This figure corresponds to Figure 6 in the original paper.

In [41], the authors present a gastric cancer segmentation method based on deformable convolution and multiscale embedding networks. The workflow of this approach is shown in Figure 6. This work combines atrous convolution, deformation convolution, and atrous space pyramid pool module. Then, the features of different semantic levels are extracted in the subsampling and feature fusion by using the lightweight decoder. Finally, intensive upsampling is performed. In this work, the dataset includes 500 pathological images of gastric cancer with an image size of2048 × 2048, and a 91.60% pixel-level accuracy and 82.65% mean intersection are achieved. This method compares with previous methods, such as FCN, VGG, U-net, and DeepLab-V3. The segmentation effect is the highest, with an accuracy rate of 91.6%.

**Figure 7:**
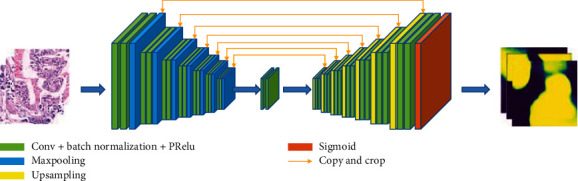
The framework of the proposed method in [42]. This figure corresponds to Figure 3 in the original paper.

In [42], a partial marker of the gastric tumor segmentation method based on reiterative learning is proposed. The architecture of the model is shown in Figure 7. In the absence of manual labeling, the average intersection of union coefficients obtained by training weakly labeled datasets is 0.883, and the average accuracy is 91.09%. After that, the deviation between patches is eliminated through overlapped region forecast.

**Figure 8:**
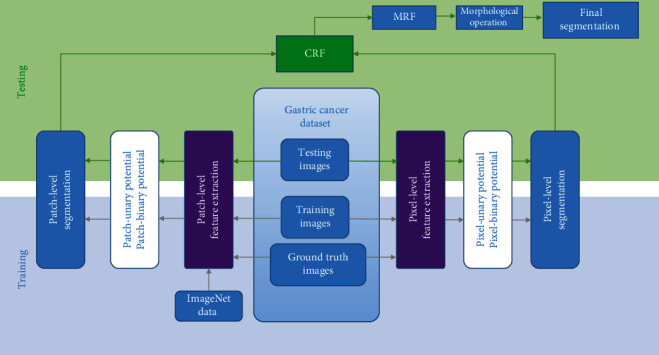
The framework of the proposed method in [43]. This figure corresponds to Figure 1 in the original paper.

In [43, 44], the authors propose a method for the segmentation of histopathological images for gastric cancer based on the hierarchical conditional random field (HCRF). The workflow of this approach is shown in Figure 8. This method can automatically locate and mark cancer regions in gastric cancer histopathological images. In this work, a total of 560 H&E-stained pathological images of gastric cancer are collected in the dataset. The segmentation accuracy is 78.91%, the recall is 65.59%, and the specificity is 81.33% through this method.

### 4.2. Summary

In summary, there are two main segmentation methods in gastric cancer histopathological image segmentation: the traditional machine learning segmentation method and the deep learning segmentation method. The traditional method uses edge detection for segmentation, and the deep learning method uses the FCN model for segmentation. U-net has a good effect on the segmentation of pathological images. However, there has been no work on the histopathological segmentation of gastric cancer using U-net. The advantage of depth segmentation lies in its good segmentation effect. It is a general frame structure and can adapt to various features. But the disadvantage is high time complexity and space complexity. Machine learning segmentation has the advantages of wide coverage and strong adaptability, while the disadvantages include large computation, complex model design, and high hardware requirements.

## 5. Classifier Design Methods

Classifier [45] design is a very important part. Choosing an appropriate classifier can make the experimental results better. In traditional machine learning, classifiers generally include SVM and RF. For deep learning networks, ResNet and U-net are commonly used networks.

### 5.1. Machine Learning Classifiers

In [27], a traditional machine learning method is presented for cell classification. This model uses a minimum-model method for multiresolution image segmentation and then extracts 7 intensity-based features, 20 morphological features, and 4 texture features. Then, the cells are divided into eight categories by Adaboost. Finally, the multiresolution segmentation results are combined and evaluated.

In [30], the authors make a comparison from three perspectives in the gastric cancer histopathological image, such as feature extraction, feature dimensionality reduction, and classifier. In classifier, this work compares RF and ANN. The ANN classifier is superior to the RF classifier.

In [32], the authors propose a method to classify histopathological images of gastric cancer by nuclear attribute relation graphs. The image is preanalyzed, and the nuclei are segmented first [46], followed by selective nuclear classification. According to the classification, different types of nuclei are constructed into cell relationship maps, and each cell relationship map is extracted for features. A total of 332 feature vectors are extracted according to the characteristics of the map, including the mean, variance, skewness, and kurtosis. Finally, random forests are used for classification.

**Figure 9:**
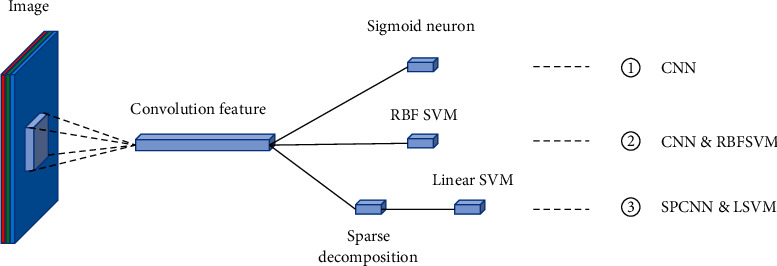
The framework of the proposed method in [47]. This figure corresponds to Figure 5 in the original paper.

In [47], the authors propose three deep learning classification algorithms for gastric cancer histopathology. The workflow of this approach is shown in Figure 9. The first group of experiments is classified by the CNN method. The first layer is the input layer, and the input image size is 512 × 512 × 3. Each layer from the second to the fourth is convolution and pooling; the convolution kernel is 3 × 3, the step length is 1, and the maximum pooling size is 2 × 2. The fifth layer is a 64 × 1 × 1 convolution feature. The accuracy of the classification results is 86.4%. In the second group of experiments, the features are extracted by CNN and then classified by an RBF kernel support vector machine. The accuracy of the classification results is 89.2%. In the third group of experiments, K-SVD is used to learn the features extracted from CNN to obtain an overcomplete dictionary, and then, sparse decomposition is carried out. Using linear kernel SVM for classification, the classification accuracy is 95%.

### 5.2. Deep Learning Classifiers

**Figure 10:**
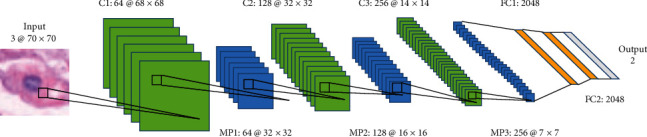
The framework of the proposed method in [22]. This figure corresponds to Figure 2 in the original paper.

In [22], a nine-layer DCNN is proposed, which is made up of three convolutional layers, three max-pooling layers, two fully connected layers, and one output layer. The workflow of this approach is shown in Figure 10. The 3 × 3 convolution kernel is convolved with a step length of 1. The convolution output is pooled to a maximum size of 2 × 2. The number of output features after three convolutional pooling is 64, 128, and 256. Finally, the pooling results are put into two full connection layers to obtain 2,048 feature vectors. This work produces an accuracy of 96.88%.

**Figure 11:**
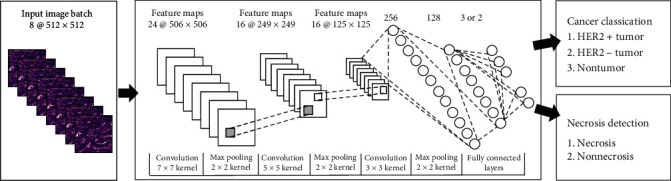
The framework of the proposed method in [23]. This figure corresponds to Figure 4 in the original paper.

In [23], the authors design a pure supervised feedforward CNN model. As shown in Figure 11, the model consists of 9 layers, 3 convolution layers and pooling layers, and finally 3 fully connected layers. The size of the convolution kernel of the three convolutional layers is 7 × 7, 5 × 5, and 3 × 3. The max pooling is the pooling layer with the size of 2 × 2. Finally, the eigenvector enters the full connection layer. The accuracy of the network in classifying tumor and necrotic areas is 69.9% and 81.4%. In addition, several comparative experiments are also done in this work. AlexNet is used in deep learning, color and texture features are used in machine learning, and RF is used for classification.

**Figure 12:**
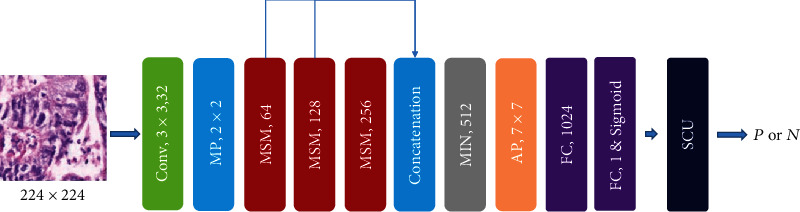
The framework of the proposed method in [24]. This figure corresponds to Figure 1 in the original paper.

In [24], the authors propose a new deep learning network-based classification model of gastric cancer histopathological images. In order to extract deep features, the deep learning network proposed in this work has different structures, namely, the shallow multiscale module and the deep network module. The workflow of this approach is shown in Figure 12. The green rectangle represents the convolutional layer, and it has a convolution kernel size of 3 × 3. The blue rectangle represents the maximum pooling layer, and the kernel size is 2 × 2. The orange rectangle represents the average pooling layer, and the kernel size is 7 × 7. The purple rectangle represents the fully connected layer. Several comparative experiments are performed, such as AlexNet, VGG-16, ResNet-50, ResNet-101, Inception-V4, and DenseNet-121. After comparison, the network of this work has achieved good results. For the patch level, the classification accuracy of the model is 97.93%. For the slice level, the classification accuracy of the model is 100%.

In [48], the authors built a 50-layer residual network model. In this model, multisize convolution kernels are used to extract features, which are 7 × 7 and 3 × 3 convolution kernels with 2 steps. Then, ReLU or Sigmoid function is used to activate the features nonlinearly. After a lot of training, the network achieves an output *F*-score of 95.5%. On top of that, the model is optimized to increase the *F*-score to 96%.

**Figure 13:**
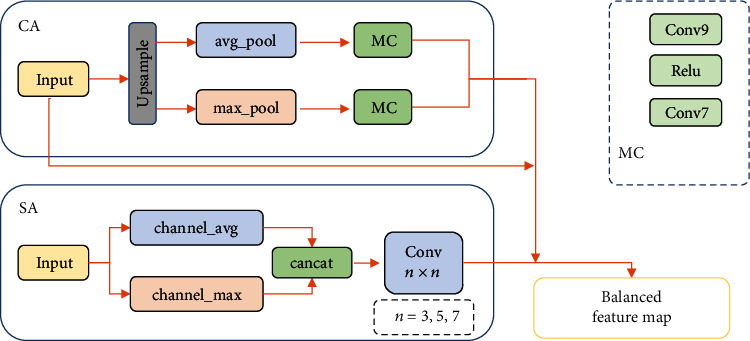
The framework of the proposed method in [49]. This figure corresponds to Figure 2 in the original paper.

In [49], a feature balanced module (FBM) is proposed that can distinguish slight differences in an image. The flowchart of FBM is shown in Figure 13. The balance module has two types of channels: the first is channel concern (CA) modules, and the second is space concern (SA) modules. In CA, the input features are upsampled, and then, max pooling and average pooling are performed. Convolution uses a9 × 9convolution kernel, ReLU function is used for activation, and convolution uses a7 × 7convolution kernel. Finally, add the two outputs as output features. In SA, the convolution kernels 3, 5, and 7 are used for convolution, and the features are compressed.

**Figure 14:**
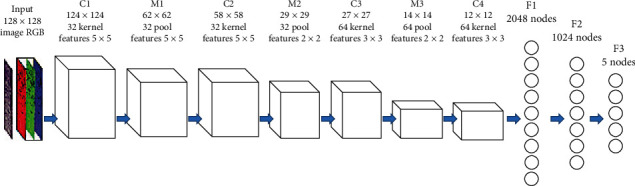
The framework of the proposed method in [50]. This figure corresponds to Figure 1 in the original paper.

In [50], the authors propose a ten-layer convolutional neural network, in which three convolutional layers extract features, four pooling layers reduce image size, and three full connection layers output feature values. The workflow of this approach is shown in Figure 14.

**Figure 15:**
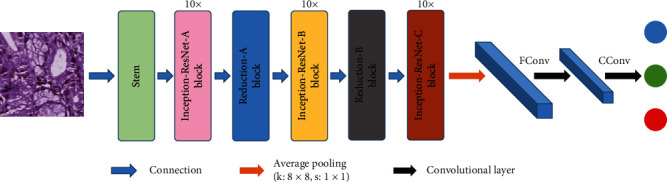
The framework of the proposed method in [51]. This figure corresponds to Figure 3 in the original paper.

In [51], the authors propose a gastric cancer histopathological image classification method based on recalibrated multi-instance deep learning. In this method, two convolution layers and one pooling layer are added to transform the ResNet-v2 network into a complete network model. The pooling layer is average pooling, and there are two convolution layers: one for feature extraction and the other for classification. This network is shown in Figure 15. The network is mainly composed of three modules, the first is local-global feature fusion, the second is the recalibration module, and the third is multi-instance pooling. The classification accuracy of the model is 86.5%.

In [52], the authors fuse the two networks of DeepLab-V3 and ResNet-50, introduce the structure of ResNet-50 network into DeepLab-V3, and build a new convolutional neural network based on DeepLab-V3. In this work, 2,166 whole slices are selected as the training set and 300 slices as the test set. After a lot of training, the final accuracy of the model is 87.3%, the sensitivity is 99.6%, and the specificity is 84.3%.

**Figure 16:**
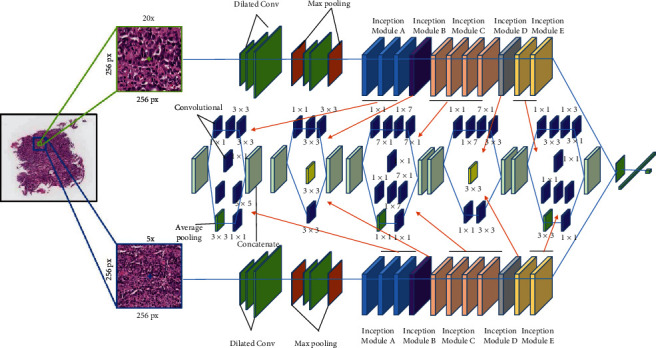
The framework of the proposed method in [53]. This figure corresponds to Figure 3 in the original paper.

In [53], the authors propose a multiscale deep learning network, in which images of different magnification levels are selected from the whole WSI image, patches of the same size are extracted from images of different magnification rates, and then these patches are put into the deep HIPO. Then, the network can learn images at multiple scales. The network is shown in Figure 16.

In [54], the authors use a standard Inception-V3 network framework. By changing the depth multiplier, the parameters are reduced. In order to increase the robustness of the image, data enhancement methods are used such as mirror and rotation. Adam optimization algorithm is used to optimize the network. After a lot of training, the network model with the smallest verification error is selected.

**Figure 17:**
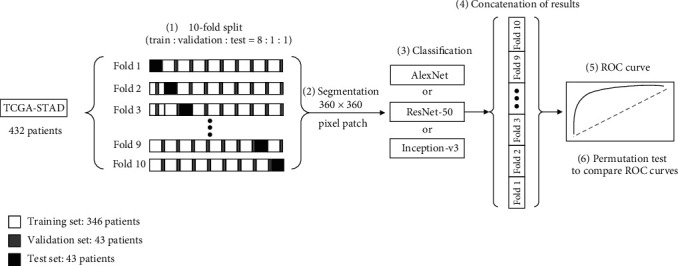
The framework of the proposed method in [55]. This figure corresponds to Figure 1 in the original paper.

In [55], the authors propose three classical convolutional neural networks for image classification: AlexNet, ResNet-50, and Inception-V3. In data selection, tenfold cross validation is used to test the performance of classification. The data is divided into ten parts, and train : validation : test = 8 : 1 : 1. For each combination, the classification results of the three classic networks are obtained. Finally, the ten results are the output in a series to calculate the accuracy, sensitivity, and specificity. The network is shown in Figure 17.

### 5.3. Summary

In the classification design of gastric cancer histopathology images, there are few articles using the traditional machine learning classification method, among which the techniques used include SVM, RF, ANN, and Adaboost. There are many classified articles using deep learning direction. Convolutional neural networks mainly include ResNet, Inception-V3, and some of their own proposed networks. In Table 2, this paper summarizes the classification methods of all gastric cancer histopathological images.

## 6. Method Analysis

### 6.1. The Gastric Histopathology Method Analysis

This paper summarizes the works on gastric cancer histopathology images from the perspectives of preprocessing, feature extraction, segmentation methods, and classifier design methods. Below, this paper briefly introduces the methods used in each step.

#### 6.1.1. Image Preprocessing Methods

By summarizing the paper, image enhancement technology is the most commonly used method in image preprocessing. Experimental training requires a large amount of data, but the data obtained from experiments are often insufficient. At this time, the experiment needs to enhance the data image.

**Figure 18:**
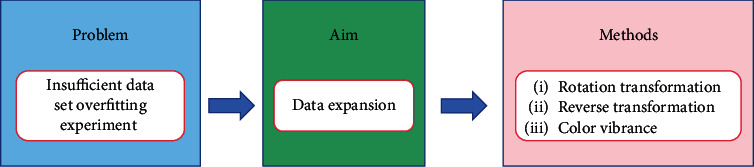
Image preprocessing method.

Figure 18 shows the preprocessing method of gastric cancer histopathological images. The work in [22, 23, 24] expanded the experimental data through data enhancement methods such as rotation and mirroring. This reduces the overfitting situation and provides a powerful help to the experiment.

#### 6.1.2. Feature Extraction Methods

**Figure 19:**
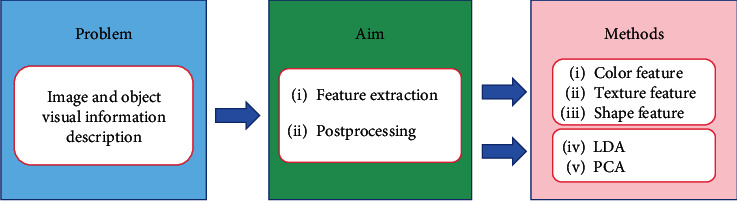
Feature extraction method.

Histopathological feature extraction of gastric cancer includes feature extraction and postprocessing of features. Feature extraction methods mainly include color, texture, and shape features. Feature postprocessing is mainly about feature dimensionality reduction.  Figure 19 shows the feature extraction method for gastric cancer histopathological images.

*(1) Traditional Feature Extraction Methods*. The main methods of color feature are RGB histograms and HSV histograms. RGB histograms reflect the composition and distribution of colors in the image, that is, which colors appear and the probability of the appearance of various colors. The work in [56] firstly proposed the method of using color histogram as the representation of image color features. HSV histogram [57, 58] is a further expression form of the RGB histogram. In RGB color space, only color information can be obtained, and the distance between colors cannot be used to represent the similarity. Therefore, features more representative of color information are extracted on the basis of RGB color space, for example, hue (H), saturation (S), and value (V). The histopathological images of gastric cancer are obtained mainly through H&E staining of gastric tissue sections. The colors of the tissues in the images obtained are in sharp contrast with each other and have relatively obvious color characteristics. The work in [23, 27] used HSV and RGB histograms to extract color features from gastric cancer histopathological images and achieved good results.

Texture feature is a kind of image global feature, which can describe the attributes of an image, mainly including HOG and GLCM. The HOG feature is a feature of statistical gradient direction change among pixels, which has good geometric invariance and optical invariance, and is excellent in human body detection. The work in [59] shows that HOG can be more effectively used in human detection than the existing feature. GLCM proposed by [60] is a matrix that describes the grayscale relationship between a certain pixel in a local area of an image and adjacent pixels or pixels within a certain distance. GLCM is a feature that describes the relationship between pixels. A gray-level cooccurrence matrix is constructed on the original image, and then, the statistical attributes in the matrix are extracted as feature vectors. Compared with normal gastric tissue cells, the texture of the cancerous stomach tissue cells changed, the nucleus became larger, and the shape of the nucleus became irregular. Extracting these texture features can distinguish normal gastric tissue from cancerous gastric tissue. In [28] [29], the HOG feature is drawn on normal, benign, and malignant gastric images to obtain the HOG feature vector.

Shape features are described by using an algorithm to get shape parameters. The cells in cancerous gastric tissues become dense, and the density between the cells can be expressed by describing the position relationship between the nuclei; thus, effective shape features can be extracted. The work [31] obtains shape features by extracting the location and structure of the nucleus. In [32], the authors extract the cell nuclei attributed relational graph of four types of cells as shape features.

*(2) Postprocessing Methods*. The dimension of feature extraction is very large in GHIA. The purpose of feature postprocessing is to reduce the dimension of features, reduce the running memory of the computer, and improve the working efficiency. The main methods include LDA and PCA. Both LDA and PCA use the idea of matrix decomposition to achieve dimensionality reduction of data.

LDA [61] is a method to realize the feature classification of two or more objects. It can be used for data dimensionality reduction or classification. It is supervised learning; LDA projects the data with a higher dimension into the vector space that can make the best discrimination. In the new vector space, the maximum interclass distance and the minimum intraclass distance of samples can be obtained. In this way, classification information and feature dimension reduction can be extracted better. The work in [29] uses LDA dimension reduction processing to get more efficient feature data.

PCA [62] is a commonly used data analysis method, which converts the original data into a set of linearly independent representations through linear transformation. It is a kind of unsupervised learning that can be used to extract the main features of the data and is often used to reduce the dimension. The work in [33] uses the dimension reduction method of PCA, and the redundant features in the original data are removed to make the variance of the projection on each dimension as large as possible.

#### 6.1.3. Segmentation Methods

**Figure 20:**
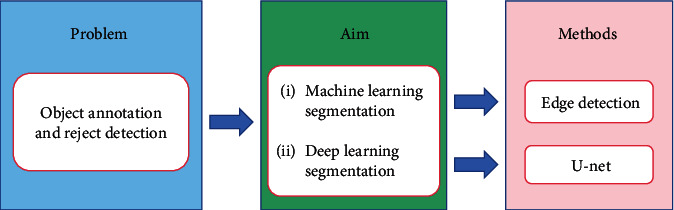
Segmentation method.

In terms of gastric cancer histopathological images, the existing image segmentation methods mainly include machine learning and deep learning. In terms of machine learning, the edge detection segmentation method is adopted, while the U-net network is used in deep learning.  Figure 20 shows the segmentation methods of gastric cancer histopathological images.

Edge detection is to find out the point where the gray level of the image changes greatly, which can reduce the amount of data, find the place with the obvious boundary, and retain the important structural attributes of the image.

U-net [63] is a semantic segmentation network based on FCN, which is originally applied to the segmentation of medical cell microscopic images. In the encoder part, the input is downsampling and downsampling through maximum pooling. In the decoder part, upsampling is carried out for the output of encoder to restore the resolution, and upsampling is implemented by deconvolution. Skip-connect is used for feature fusion. The work in [41] uses U-net to segment gastric histopathology images and to compare with other neural network structures. U-net has a better segmentation effect.

#### 6.1.4. Classifier Design Methods

**Figure 21:**
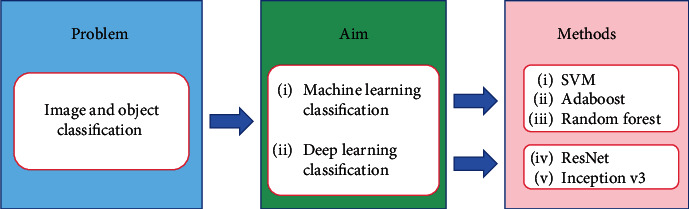
Classifier design methods.

In the gastric histopathology images, there are two kinds of classification designs, machine learning and deep learning. Machine learning classifier mainly uses SVM, Adaboost, and random forest. Deep learning uses convolutional neural networks for classification, such as ResNet and Inception-V3.  Figure 21 shows the classifier design methods of the gastric cancer histopathological images.

*(1) Machine Learning Classifier Design Methods*. SVM [64] is a dichotomous model. By mapping data to a high-dimensional space and finding a hyperplane in the high-dimensional space, the distance between separated data classes is maximized, so as to achieve the purpose of optimal classification. This work [48] uses the SVM classifier and optimizes kernel function in the SVM classifier to improve classification accuracy.

The Adaboost [65] algorithm is a lifting method, which combines several weak classifiers into a strong classifier. Adaboost trains one weak classifier at a time and then iterates to train the next weak classifier after training. The work in [27] uses the Adaboost classifier to classify three feature groups of color, texture, and shape and finally obtains the optimal classification result.

Random forest [66] is an ensemble learning method in machine learning. This method integrates multiple decision trees through ensemble learning. Limitations can be avoided by integrating multiple models. This work [32] uses a random forest classifier to classify the shape features of the reticular map made up of nuclei.

*(2) Deep Learning Classifier Design Methods*. ResNet [67] applies the idea of residuals to the convolutional neural network. Different from the traditional convolutional neural network which directly represents the mapping relationship between input and output, it represents the residual between input and output through multiple parameterization layers. The authors in [48, 55] use an encoder based on ResNet to process the gastric histopathological image. Compared with other neural network structures, ResNet can provide end-to-end services for users and retain more detailed information on gastric cancer histopathological images.

Inception-V3 [68] uses convolutional kernels of different sizes in the convolutional layer to improve the perception of the network and proposes batch standardization to alleviate the problem of gradient disappearance. It uses better convolution kernel decomposition methods on the basis of Inception-V2 to make computational complexity more efficient, reduce representational bottlenecks of features, and avoid information loss. The work [54] has optimized the Inception-V3 network architecture to reduce network parameters, avoid overfitting, and improve network effectiveness.

### 6.2. The Potential Methods in Gastric Histopathology Image Analysis

#### 6.2.1. Image Preprocessing Methods

In image preprocessing [69], methods in other fields can also be applied in the field of gastric cancer histopathology.

In [70], the authors develop an image denoising network based on CNN and optimize the denoising network by utilizing the advantages of complex numerical operations to increase the tightness of convolution.

In [71], the authors propose a new image denoising method using a new loss function, which pays attention to the perceived visual quality and applies the loss function to the jump connection network to obtain images with high precision details. In the histopathological images of gastric cancer, the operation of sectioning and staining will affect the image quality and generate noise. The image denoising method in [70, 71] can be applied to the histopathology of gastric cancer to improve the image quality.

In [72], the authors propose a data expansion method. In the histopathology of gastric cancer, due to the problem of data collection, the amount of data is relatively small, so this data expansion method can be applied to the histopathology of gastric cancer to increase the dataset and improve the experimental quality.

In [73], the authors propose a method for contrast enhancement of retinal images, which uses a shearlet transform and adaptive gamma correction-based singular value equalization hybrid technique combined with a contrast-limited adaptive histogram equalization technique. In the histopathological images of gastric cancer, HE staining is usually used in the image, which has distinct color features. The image contrast enhancement technology in [73] can make the color features more prominent and improve the image quality.

#### 6.2.2. Feature Extraction Methods

The work in [74] summarizes the feature extraction methods. Feature extraction is also an important step in gastric cancer histopathological images, and many feature extraction techniques in [74] can be applied to gastric cancer histopathological images.

In [75], the authors extract image feature vectors based on computed radiography and obtain 279 image descriptors. Through the Hellwig method, they find the feature combination set of the overall index with the maximum information capacity. Then, by testing the classification results of 11 classifiers, they select the two feature sets with the highest accuracy. This method can also be applied to the feature selection of gastric cancer histopathological images to select the most effective feature.

In [76], the authors propose a new unsupervised feature selection method, which calculates the dependencies between features and avoids selecting redundant features. The experimental operation efficiency is improved. In the histopathological images of gastric cancer, the experimental data are large, the system execution time is long, and the memory demand is large during the experiment. The feature selection method mentioned in [76] can also be applied to the histopathology of gastric cancer to improve the operating environment of the system and the operation efficiency of the experiment.

#### 6.2.3. Segmentation Methods

The work in [77] summarizes the segmentation techniques applied in various fields. In the histopathology of gastric cancer, image segmentation is often used, and the current mainstream segmentation techniques can also be applied in the histopathology of gastric cancer.

In medical images, the authors in [78] propose a new polyp segmentation method based on multidepth codec network combination. The network can extract the features of different effective receptive fields and multisize images to represent the multilevel information, and it can also extract the effective information features from the missing pixels in the training stage. The method of [78] can be applied to gastric cancer histopathological images,

In [79], the authors propose an image segmentation method based on a scalable multichannel weighted region model. In order to improve the performance of image Mosaic, a new edge detection function is proposed. The model can also be used to segment images in gastric cancer histopathology.

In [80], the work proposes a radiology-based deep supervised U-net for the segmentation of prostate and prostatic lesions. The U-net network can also be used to segment the normal and cancerous areas in the histopathological images of gastric cancer.

In [81], the work proposes an image segmentation method combining low-level operation, affine probabilistic graph, and multigraph. This method is used for segmentation of computed tomography (CT) images. Experimental results show that compared with atlas selection and nonrigid registration, this method has better performance in the whole region of interest. This method can also be applied to histopathological image segmentation of gastric cancer and obtain good results.

#### 6.2.4. Classifier Design Methods

In [82], the authors summarize various classifier techniques, many of which can be applied to gastric cancer histopathological images.

In [83], the authors used a combination of convolutional neural network and machine learning classifier to classify traffic density and compared CNN, CNN-SVM, CNN-RF, and CNN-Xgboot to select an appropriate model. This method can also be applied to gastric cancer histopathological images. Features are extracted through deep learning network and then classified by machine learning method.

In [84], the authors propose a new method for classification of breast masses based on the deep learning model. It can automatically process small two-dimensional radiofrequency signals and their amplitude samples. A deep learning classification model of breast masses was constructed using radiofrequency data. This classification model can also be used for the classification of gastric cancer pathology.

In [85], the authors propose a Deep CNN based on the Dolphin Echolocation-based Sine Cosine Algorithm. The algorithm uses Dolphin-SCA-based fuzzy fusion model for segmentation, and then, the statistical attributes of weight, mean value, variance, and skewness are used for feature calculation. Finally, the convolutional neural network is used for classification. This method can also be applied to the histopathology of gastric cancer.

In [86], the authors propose a dictionary learning method based on multiclass loss feedback discrimination for support vector machines. The framework learns the discriminant dictionary while training the SVM, which makes the features extracted from the learner dictionary better matched with the SVM. This classification model can also be used for the classification of gastric cancer pathology.

### 6.3. The Gastric Histopathology Methods for Other Potential Fields

#### 6.3.1. Image Preprocessing Methods

In the histopathology of gastric cancer, the image preprocessing method is mainly data enhancement. The data enhancement techniques can apply to other areas, such as database enhancements in the field of microbiology. In [87], the microbial data are expanded by combining geometric transformation and GAN network. In the pathological images of breast cancer, the number of datasets is also insufficient. The data enhancement technique is also applicable to increase the dataset to make the experiment more accurate. In [88], the authors use the geometric transformation method to increase the number of datasets, reduce the overfitting situation, and make the experiment more accurate.

#### 6.3.2. Feature Extraction Methods

In the histopathology of gastric cancer, CRF and GLCM methods are used for feature extraction. These methods are also widely used in the field of microbiology because the cell morphology and size of gastric cancer histopathology are similar to that of microorganisms. In [89], a CNN-CRF network framework is adopted to extract features, which CRF is used for feature postprocessing. Texture feature is also a common feature extraction method in breast cancer pathological images. In [90], the GLCM method is used for feature extraction.

#### 6.3.3. Segmentation Methods

In gastric cancer histopathology, image segmentation includes threshold segmentation based on color and edge detection based on texture. Compared with the gastric cancer histopathological image, the color contrast between the microorganisms and the background in the microbial image is also relatively bright, and threshold segmentation can be adopted. The microbe's texture and background are also quite different, so edge detection can be used. In [91], the authors use an image segmentation method related to color and texture. In the pathology of cervical cancer [92], because cervical cancer cells are similar to gastric cancer cells, the segmentation method of gastric cancer can also be used in the pathological images of cervical cancer, and the minimum-model method can also be used in cervical cancer.

#### 6.3.4. Classifier Design Methods

In the histopathology of gastric cancer, SVM and RF methods were used for image classification. These methods can also be applied to microbial images and pathological images of cervical cancer. In [93], the methods of microbial classification are summarized and it is found that the methods applied to gastric cancer histopathological images can be applied to microbial images. In [94], SVM classifier is used to classify cervical cancer.

## 7. Conclusion and Future Work

This paper reviews the methods of histopathological image analysis of gastric cancer, including preprocessing, feature extraction, segmentation, and classification. In preprocessing, in order to solve the problem of lack of data, the method of data enhancement is adopted. The data enhancement method is mainly image rotation and geometric transformation. In terms of feature extraction, it is summarized from two aspects: the machine learning method and the deep learning method. The machine learning method includes color feature, texture feature, and morphological feature. In terms of image segmentation, there are many papers adopting the machine learning method, mainly adopting edge detection, the segmentation method, and the U-net convolutional neural network for deep learning. In terms of image classification, machine learning classifiers such as SVM and RM are mainly applied. Deep learning networks employ some classical convolutional neural network structures, such as ResNet and Inception-V3. Others are frameworks combining machine learning and deep learning, extracting features by deep learning network, and then classified by machine learning classifier.

In the future, there is still space for improvement in the histopathological image analysis of gastric cancer. First of all, there are few papers related to image analysis methods in the field of gastric cancer. Researchers can develop a new network model combining with gastric cancer histopathological images to analyze gastric cancer histopathological images. The following techniques, for example, “DoDNet: Learning to Segment Multiorgan and Tumors from Multiple Partially Labeled Datasets” [95], “3D Cascaded Convolutional Networks for Multivertebrae Segmentation” [96], and “PGL: Prior-Guided Local Self-supervised Learning for 3D Medical Image Segmentation” [97], can be applied to the pathological image processing of gastric cancer. Secondly, in the field of gastric cancer histopathology, there is a lack of complete, clear, and accurately labeled pathological images, so the establishment of more perfect data can provide great help for the experiment. Finally, in terms of feature extraction and classifier design, there are many novel techniques that can be applied to gastric cancer histopathological image analysis, which is a promising and valuable research direction.

## Figures and Tables

**Table 1:** Summary of feature extraction.

Aim	Year	Reference	Team	Method
Feature extraction	2017	[23]	Sharma et al.	GLCM, LBP, HSV, and RGB
	2015	[27]	Sharma et al.	RGB feature, shape feature, and texture feature
	2017	[28]	Korkmaz et al.	HOG feature
	2017	[29]	Korkmaz et al.	HOG feature
	2018	[30]	Korkmaz et al.	LBP feature, HOG feature
	2013	[31]	Cosatto et al.	Feature of nuclear location relationship
	2017	[32]	Sharma et al.	Feature of nuclear location relationship

Postprocessing	2017	[29]	Korkmaz et al.	LDA
	2018	[30]	Korkmaz et al.	SNE, Isomap, MDS, LLE, LDA, and T-SNE
	2012	[33]	Gan et al.	PCA and LDA

**Table 2:** Classification-based gastric cancer image analysis.

Aim	Year	Reference	Team	Dataset	Method	Evaluation
Machine learning	2015	[27]	Sharma et al.	5,541 30x and 3,730 40x images	Adaboost	ACC = 59.15%
	2017	[32]	Sharma et al.	795 images	Random forest	ACC = 73.78%
	2018	[47]	Liu et al.	560 cancer and 140 noncancer	CNN and SVM	ACC = 95%

Deep learning	2017	[22]	Garcia et al.	3,275 images	DCNN	ACC = 96.88%
	2017	[23]	Sharma et al.	21,000 images	Proposed CNN	Accuracy of cancer classification is 69.9%; accuracy of necrosis detection is 81.4%
	2018	[24]	Li et al.	560 cancer and 140 noncancer	Proposed CNN	Accuracy of patch level classification is 97.93%; accuracy of slice level classification is 100%
	2018	[48]	Liu et al.	1.2 million images	ResNet	*F* − score = 96%
	2019	[51]	Wang et al.	608 whole slide images	ResNet	ACC = 86.5%
	2020	[52]	Song et al.	2,123 digital slides	DeepLab-V3 and ResNet	Acc = 87.3%, Sn = 99.6%, Sp = 84.3%
